# A chewable pediatric preparation of ibuprofen is palatable and acceptable to children

**DOI:** 10.1002/pne2.12013

**Published:** 2020-02-10

**Authors:** Steven Barnett, Aomesh Bhatt

**Affiliations:** ^1^ Reckitt Benckiser Healthcare Hull UK; ^2^ Reckitt Benckiser Research & Development Berkshire UK

**Keywords:** analgesia, ibuprofen, paediatric medicine, palatability

## Abstract

The development of palatable and acceptable analgesics for children is a major challenge. Given the majority of medications are administered orally, and children are more sensitive to and less tolerant of bitterness, novel “child‐friendly” preparations need to be developed and tested specifically in this patient population. This study investigated the palatability and acceptability of a therapeutic dose of ibuprofen in the form of soft chewable capsules in 100 healthy children aged 7‐12 years of age and the acceptability of this novel preparation to their caregivers. About 97% of children adhered to a full therapeutic age‐related dose, with 72% of these participants rating the preparation as acceptable on a hedonic facial scale. Despite 22% of children noting a “hot, spicy, or burning” sensation, consistent with known chemesthetic effects of ibuprofen, 83% of children confirmed they would take the medication in future, which rose to 87% in the context of future illness. In addition, after observing their children ingesting the medication, 92% of parents/guardians confirmed that they would be happy to administer this preparation of ibuprofen to their child if they were unwell. In conclusion, ibuprofen administered in the form of soft chewable capsules was palatable and acceptable to the majority of children and their parents/guardians and may provide a convenient and easy to dose preparation to reduce fever and relieve pain in children.

## 1 BACKGROUND

Palatability is a major obstacle to compliance of medicines in children,^1^ and it has therefore become a primary consideration in the development of novel pediatric formulations.^2^, ^3^, ^4^ Both parents and pediatricians recognize that taste is a common reason for medication refusal in children,^5^ and therefore, “child‐friendly” preparations must be developed and specifically tested in this patient population.^6^

Ibuprofen is increasingly purchased by parents over the counter for the management of pain and fever.^7^ Unfortunately, like many drugs such as paracetamol and common antibiotics,^8^ ibuprofen has a characteristically bitter taste and can cause irritation to the throat when ingested orally.^9^ Taste perception and tolerance develop during childhood and young children are particularly sensitive and averse to bitterness.^10^, ^11^, ^12^ While this may serve an adaptive evolutionary benefit in promoting the avoidance of toxic substances,^13^ it presents a considerable barrier to the administration of medications such as ibuprofen.

Improved palatability of pediatric medications has been associated with enhanced adherence.^14^ For adults and older children, the unpleasant taste or bitterness of active ingredients and excipients of drugs can be masked in coated tablets or capsules that can be swallowed. Such preparations are, however, unsuitable for some young children, and medications are commonly administered in liquid form, which also allows flexibility for age‐ and weight‐appropriate dosing.^15^ However, bitterness remains an issue and is challenging to mask.^16^ Modern masking techniques include bitter receptor antagonists, sweeteners, flavorings, and chemical modification of the active pharmaceutical ingredient.^17^ Chewable formulations have more recently been developed, which have the advantages of precise pediatric dosing, stability, convenience of storage and transport, and simplicity of delivery.^18^ Some studies have suggested chewable capsules may be preferred by school children, adolescents, and their caregivers.^19^ However, acceptability of this dosage form has not been extensively evaluated in children.^20^ This study investigated the palatability and acceptability of a soft natural orange‐flavored chewable formulation of ibuprofen (Chewel^®^) in healthy children and the acceptability of this novel preparation to their caregivers.

## 2 METHODS

### 2.1 Aims

The primary aim of this study was to investigate the acceptability of ibuprofen soft chewable capsules to healthy children aged 7‐12 years using a hedonic facial scale and questionnaire. The acceptability of this preparation to caregivers was also assessed as a secondary outcome.

### 2.2 Study design and participants

An open‐label single‐center taste‐testing study of a single age‐related dose of ibuprofen soft chewable capsule in healthy children was conducted.

Each ibuprofen soft chewable capsule consisted of an orange‐flavored, square, gelatine chewable capsule, containing 100 mg of ibuprofen, manufactured, and supplied by Banner Pharmacaps Europe. Ibuprofen Chewels^®^ was developed using Banner's (now Patheon's) proprietary technology platform (patent US 8,097,279 B2). They contain an easy‐flowing liquid suspension of Ibuprofen that turns semi‐solid upon cooling to room temperature, which is taste masked with a combination of flavors, sweeteners, and flavor enhancers. The ibuprofen is encapsulated within a plasticized gelatine shell, providing a chewable capsule with a soft gummy texture.

Participants were healthy children aged 7‐12 years who were recruited from a volunteer database and poster advertising. Each child's eligibility was assessed by an investigator according to exclusion criteria during a screening visit, and compliance with additional restrictions for taste testing was verified on the day of study (Table 1).

**Table 1:** Study exclusion criteria

Screening exclusion criteria
Intercurrent respiratory infection
Drug sensitivity or allergy
Hepatic or renal impairment
Asthma
History of gastrointestinal disorder including (ulcers and bleeding)
>2 siblings enrolled in the study
Relative of investigator
Participant in taste‐testing study in previous 72 h
Participant in clinical trial of an IMP in previous month
Restrictions for taste testing (on day of study)
Prescription medication within 7 d of study
Analgesics on day of study
Food/drink likely to alter taste (spice/menthol) on day of study
Nonprescription medication 4 h before study

Written informed consent was obtained from the caregiver in addition to written assent from each child. The study was approved by Reading Independent Ethics Committee and conducted in accordance with the Declaration of Helsinki, as referenced in EU Directive 2001/20/EC. It complied with International Conference on Harmonisation (ICH) Good Clinical Practice (GCP) and applicable regulatory requirements.

### 2.3 Study procedure

Children were recruited to the study between September and October 2012. Following palate cleansing with water and water biscuits, children aged 7‐9 years received 200 mg Ibuprofen (in the form of 2 chewable capsules), and children aged 10‐12 years received 300 mg ibuprofen (in the form of 3 chewable capsules), in accordance with guidance from the British National Formulary for children (BNFc).

Palatability of the drug was assessed on completion of the full dose or at the point of refusal using a 7‐point hedonic facial scale, whereby children were asked to select a face reflecting their opinion of the medicine. The investigator then conducted an interview using a predefined questionnaire that explored whether the child had a desire to remove the medication from their mouth, their willingness to take the medicine in future, willingness to take the medicine if unwell in the future, description of the flavor, likes and dislikes about the flavor, the presence of an aftertaste, sensations in the mouth and throat, presence of tingling, and the acceptability of the size of the ibuprofen soft chewable capsules. Parents were also asked whether they would administer the medicine in future if their child was unwell and to grade the appeal of a medicine that is easy to dose, does not require water, and can be administered easily.

Although safety was not formally assessed during the study, the supervising investigator recorded all adverse events experienced by children during the study visit and reported their severity and likely relationship to the drug.

### 2.4 Outcomes

The primary endpoint of the study was the percentage of children who rated a full dose of ibuprofen soft chewable capsules as “acceptable,” with acceptability defined as a score greater than four on a 7‐point hedonic facial scale. The overall scores from children consuming at least one chewable capsule were assessed as a secondary endpoint. Further acceptability variables were derived from the subject questionnaire, which included the percentage of children who would take the medication if unwell and the percentage of parents who would administer the medication if their child were unwell. The results are presented as percentages and mean (SD, standard deviation) where appropriate.

## 3 RESULTS

Between September 24, 2012, and October 16, 2012, 105 children were screened. Following the exclusion of five children (three children due to a health conditions and two due to concurrent medications), 100 healthy children were enrolled in the study (Table 2). About 50% of children were aged 7‐9 years, and 50% were aged 10‐12 years, with an overall mean age of 9.4 years. Sixty‐one children were male (61%) and 39 (39%) female. Nine children had ongoing medical conditions, which included eczema, hay fever, and migraine/abdominal migraines. Six children reported previous medical conditions or surgeries, which included Henoch‐Schonlein purpura, tonsillectomy, bilateral T‐tube insertion, an adenoidectomy and grommets insertion, and grommet insertion (2 children). There were no deviations from exclusion or inclusion criteria.

**Table 2:** Participant demographics

Demographic	n = 100
Age mean (SD)	9.4 (1.7)
Age Groups (%)	
7‐9 y	50
10‐12 y	50
Sex (%)	
Male	61
Female	39
Ethnicity (%)	
Caucasian	99
Asian	1
Ongoing medical conditions (%)	9
Previous surgery (%)	5

All children ingested at least one chewable capsule and selected a face on the 7‐point hedonic scale. Ninety‐seven out of the 100 enrolled children (97%) completed a full age‐related therapeutic dose. Although no subjects were withdrawn from the study, one 8‐year‐old girl refused her second chewel and two children aged 10 (1 girl and 1 boy) refused their third chewel.

Of the 97 children who completed a full dose, 72% (70/97) selected a score greater than 4 on the hedonic facial scale, satisfying predefined acceptability criteria. This included 71% of children aged 7‐9 years (35/49) and 73% of children aged 10‐12 years (35/48), and 75% (45/60) of male, and 68% (25/37) of female participants. Overall, 71 of the 100 participants (71%) who consumed at least one chewable capsule rated the medicine acceptable, with a mean score of 5.2 (SD 1.6).

On direct questioning, 83% of participants confirmed they would take the medicine again. Of the children who would refuse the medication in future, 8 attributed this to the taste, 4 ascribed this to the medication being “hot,” “burning,” or “spicy,” and 4 children felt it induced coughing or hurt their throat. About 20% of children confirmed having had a desire to remove the medication from their mouth due to the taste, with five children attributing this to a sore or burning throat and five referring to the medication as “spicy.” About 87% of children, however, confirmed that they would take the chewable capsule to aid their recovery from illness and 8% gave a neutral response.

Regarding the flavor of the medicine, 64% of children correctly described an orange flavor and persistence of a flavor after swallowing was noted by all but two children. About 63% of children described it as mild, with 43% continuing to ascribe a fruit or sweet flavor and 9% describing the aftertaste as “hot, spicy, or strong.” When asked to consider the effect of the medication on their mouth and throat, 64% of children confirmed a “tingling” sensation. However, 43% described a positive feeling or no effect in their mouth and 52% described a positive feeling or no effect in their throat. Nevertheless, a “spicy,” “hot,” or “burning” sensation was noted by some of children in their mouth (22%) or throat (15%). About 89% of children approved of the size of the chewable capsule.

After observing their children ingesting the medication, 92% of parents/guardians confirmed that they would be happy to administer this preparation of ibuprofen to their child if they were unwell. About 5% of caregivers were concerned that their child disliked the taste of the medication, and one caregiver was discouraged by their child's coughing. All parents, however, confirmed that the concept of a chewable medication that is easy to dose and does not require water is appealing. Additionally, parents volunteered positive comments regarding the portability, convenience of dosing, size of the dose form, and the avoidance of spillage.

Twenty‐six mild adverse events were reported by an investigator as probably related to the medication during the study. These events consisted of eye watering and a sore throat in two children, respectively, and coughing during or after consumption of the chewable capsules in the remaining cases. These events occurred more frequently in children aged 10‐12 years (34%) than children aged 7‐9 years (16%).

## 4 DISCUSSION

Palatability is a fundamental priority in children's medicines considering its significant impact on adherence. Considering children may perceive tastes differently to adults,^5^ palatability studies of pediatric medications must be performed in children rather than healthy adults and employ age‐appropriate validated scales in order to be informative. In this study, ibuprofen was administered to healthy children aged 7‐12 years in the form of soft chewable natural orange‐flavored capsules and overall was palatable and acceptable to most children and caregivers. Almost all children adhered to a full therapeutic dose, and nearly, three‐quarters of participants rated the medication as acceptable on a hedonic scale. The majority of children responded positively to the flavor and size of the chewable tablets, and most children were not concerned by the persistence of a commonly described fruity flavor after swallowing. Importantly, the majority of the children (83%) confirmed that they would take the analgesic in future, and this interestingly rose to 87% when children were asked to consider their compliance in the context of illness. The acceptability of this formulation was further supported by 92% of caregivers who confirmed that they would happily administer this preparation of Ibuprofen to their child in the event they were unwell.

Studies have previously suggested differences in taste preferences with age and sex in children.^21^, ^22^, ^23^ However, in this study no difference in acceptability was observed between the two age groups or any substantial difference noted between the sexes. Although 83% of children highlighted a positive response regarding the flavor of the medication, 16% of subjects disliked the chewable tablets as they perceived them as being hot, spicy, or too strong. Exaggerated age‐related changes in taste sensitivity have been reported in children with particular bitter receptor genotypes^22^ and may account for some of the variation in sensitivity to bitterness observed in this study. Tingling or burning of the throat or mouth was commented on by less than 25% of children, and coughing was observed by an investigator in 23% of children. These effects were not unexpected as irritation of the throat has been reported following ingestion of other preparations of ibuprofen.^9^ However, despite these effects, the majority of children expressed a willingness to take the medication in the context of future illness, suggesting overall acceptability of the medication.

Consistent with a previous review of chewable medicines for children,^18^ the medication in this study was generally well tolerated and safe, with no incidences of choking. Furthermore, caregivers highlighted precise dosing, portability, and ease of delivery as advantages of this formulation. The general appeal of this preparation of ibuprofen was further evidenced by several parents (3%) volunteering concerns that children could ingest chewable tablet mistakenly as sweets. This highlights the importance of safe storage of medicines in the home, particularly in the case of “child‐friendly” preparations that are palatable to children.

It has taken over 6 years from the initial study recruitment to obtaining the initial marketing authorization of the medicine (Nurofen for children 100 mg, chewable capsules, soft) in the UK which was granted in August 2018.

In conclusion, ibuprofen administered in the form of a soft chewable orange‐flavored chewable tablet in this study was palatable and acceptable to the majority of children and their parents/guardians. Chewable capsules (Chewels^®^) could provide a convenient, easy to dose, and acceptable form of ibuprofen, in which parents could use for pain relief or fever reduction in children, decreasing the risk of medication refusal or potential misdosing. Further studies are needed to establish the equivalence or superiority of this formulation of ibuprofen to commonly administered liquid preparations in terms of palatability and acceptability.
